# Postoperative Gait Performance Following Pertrochanteric Fractures Is Influenced by the Preoperative Condition of the Gluteal Muscles

**DOI:** 10.7759/cureus.68176

**Published:** 2024-08-30

**Authors:** Mitsuaki Noda, Shunsuke Takahara, Atsuyuki Inui, Keisuke Oe, Shin Osawa, Takehiko Matsushita

**Affiliations:** 1 Department of Orthopaedics, Himeji Central Hospital, Himeji, JPN; 2 Department of Orthopaedics, Hyogo Prefectural Kakogawa Medical Center, Kakogawa, JPN; 3 Department of Orthopaedic Surgery, Kobe University Graduate School of Medicine, Kobe, JPN; 4 Department of Orthopedics, Kobe University Graduate School of Medicine, Kobe, JPN

**Keywords:** postoperative, cross-sectional area, body mass index: bmi, gluteus medius, density, computed tomography, gluteal muscles, pertrochanteric fractures, gait

## Abstract

Introduction

Amid an increasing number of patients with pertrochanteric fractures, early prediction of postoperative gait potential could reduce unnecessary rehabilitation and hospitalization. The relationship between preoperative gluteal muscle condition and postoperative gait outcomes remains unclear. The gluteal muscles are crucial for mobility, and their cross-sectional area (CSA) and fatty infiltration are indicators of physical function. Preoperative computed tomography (CT) provides quantitative data on muscle CSA and density, measured in Hounsfield Units (HU). This study aimed to identify which preoperative muscle index, CSA, BMI-adjusted CSA, or muscle density, best predicts postoperative gait ability. We hypothesized that a higher adjusted CSA and muscle density in the gluteus muscles would be associated with superior gait performance.

Materials and methods

Preoperative assessments included radiographs and CT scans. Patients under 75, with less than four weeks of follow-up, prior contralateral hip surgery, prefracture immobility, male patients, high-energy trauma, or conditions impairing physical performance were excluded. Gait performance was evaluated four weeks postoperatively, classifying patients into two groups: the 'parallel bar group,' requiring parallel bars, and the 'walker group,' walking independently. Patients underwent CM nailing. Preoperative CT scans measured the CSA and muscle density of the gluteus maximus and medius. Measurements were taken from the non-injured side to avoid interference from the fracture. Muscle borders were manually traced, and the CSA and muscle density in HU were calculated.

Results

Out of 81 patients, 49 met the inclusion criteria (mean age: 87). The patients were divided into the ‘parallel bar group’ (n=19) and the ‘walker group’ (n=30) based on postoperative gait performance. No significant differences in age, weight, height, or fracture laterality were observed between groups.

The mean (and standard deviation (SD)) of CSA for the gluteus maximus in the 'parallel bar group'/in the 'walker group' was 2211.8 ± 469.8 mm²/2440.0 ± 586.2 mm², respectively (p=0.15), and for the gluteus medius, it was 1751.7 ± 415.2mm²/1869.1 ± 448.3mm², respectively (p=0.36). The mean (and SD) muscle density for the gluteus maximus in the 'parallel bar group'/in the 'walker group' was 13.6 ± 12.7 HU / 20.6 ± 13.0 HU (p=0.07), and for the gluteus medius, it was 25.2 ± 8.4 HU/31.8 ± 10.1 HU, respectively (p=0.02). The ROC curve identified a 30.9 HU cut-off for gluteus medius density, with sensitivity and specificity of 60.7% and 78.9%.

The mean (and SD) of BMI-adjusted CSA for the gluteus maximus in the 'parallel bar group'/ in the 'walker group' was 116.4 ± 26.8 m²/10^6^ kg/124.3 ± 29.2 m²/10^6^ kg, respectively (p=0.35), and for the gluteus medius, it was 93.3 ± 27.2 m²/10^6^ kg/95.4 ± 21.3m²/10^6^kg, respectively (p=0.78).

Conclusion

This study analyzed preoperative CT images of women aged 75 and older with pertrochanteric fractures, comparing gluteal muscle CSA and density with postoperative walking ability. The gluteus medius density was significantly higher in the superior gait group, with a cut-off value of 30.9 HU. However, no significant differences were found in the gluteus maximus density, CSA, or BMI-adjusted CSA. These findings partially support the hypothesis, emphasizing the importance of muscle evaluation in predicting postoperative gait performance.

## Introduction

Pertrochanteric fractures are among the most common injuries treated in the elderly population. With an estimated six million cases globally by 2050 [1], the need for sustainable medical resources is critical [2]. When patients fail to regain independent gait postoperatively, they are often required transfer to a nursing home after months of rehabilitation [3]. Accurate prediction of postoperative gait potential upon admission could prevent unnecessary prolonged rehabilitation and hospitalization, benefiting both national healthcare expenditure and the patients and their families [4].

Currently, the relationship between preoperative gluteal muscle condition and postoperative gait outcomes in patients with pertrochanteric fractures is not well understood. The gluteal muscles, essential for functional mobility, can reflect the extent of functional impairment [5]. The muscle cross-sectional area (CSA) is a well-established indicator of physical activity progression [5,6]. Additionally, fatty infiltration within muscles is associated with reduced lower extremity performance and mobility limitations in older adults [7]. As intramuscular fatty degeneration increases, mobility decreases [8,9]. Preoperative computed tomography (CT) is commonly used to evaluate gluteal muscles, providing quantitative data on muscle CSA and quality, including muscle density, expressed in Hounsfield Units (HU) [10].

In the present study, we compared three preoperative indices, CSA, BMI-adjusted CSA, and muscle density in HU, for the gluteus muscles between patients with high and low gait performance at four weeks postoperatively. The primary aim was to identify which muscle index most accurately predicts postoperative gait ability. We hypothesized that a higher adjusted CSA and muscle density in the gluteus muscles would be associated with superior gait performance.

## Materials and methods

Patient data sources

Geriatric patients (aged 75 years or over) with pertrochanteric fractures requiring surgical intervention, treated between March 2018 and January 2024, were retrospectively identified from a trauma database at Nishi Hospital in Kobe, Japan. To be included in the study, patients needed to have complete data sets, including preoperative two-directional plain radiographs (anteroposterior and lateral views), 3D CT scans, and demographic information. Additionally, the fractures were restricted to those caused by low-energy trauma, such as falls from a standing height. Exclusion criteria included: (1) patients under 75 years of age, (2) follow-up less than four weeks, (3) history of contralateral hip surgery, (4) prefracture inability to walk, (5) male patients, too small in number to conduct meaningful comparative analysis, (6) high-energy trauma such as traffic accidents or falls from height, and (7) coexisting conditions that could substantially impair physical performance, such as significant neuropathy of the major nerves or spinal cord.

This study was approved by the Nishi Hospital Ethics Committee, with informed consent obtained from each patient or their representative (approval number: 2021-1), and the approval date was February 7, 2021.

Research outcomes and assessments

Gait performance was assessed four weeks postoperatively based on the walking aid required. Patients were classified into two groups: the ‘parallel bar group,’ where participants needed a parallel bar for walking or standing, and the ‘walker group,’ where participants walked independently with or without a walker. Gait activity levels were extracted from medical records maintained by physicians, nurses, and physical therapists. In cases where the exact ambulatory level at four weeks was not recorded, the data were estimated from records taken between 3 and 5 weeks postoperatively.

Surgical procedure

All patients underwent cephalomedullary nailing using a similar technique [11,12].

CT measurements for muscular indices

**Figure 1:**
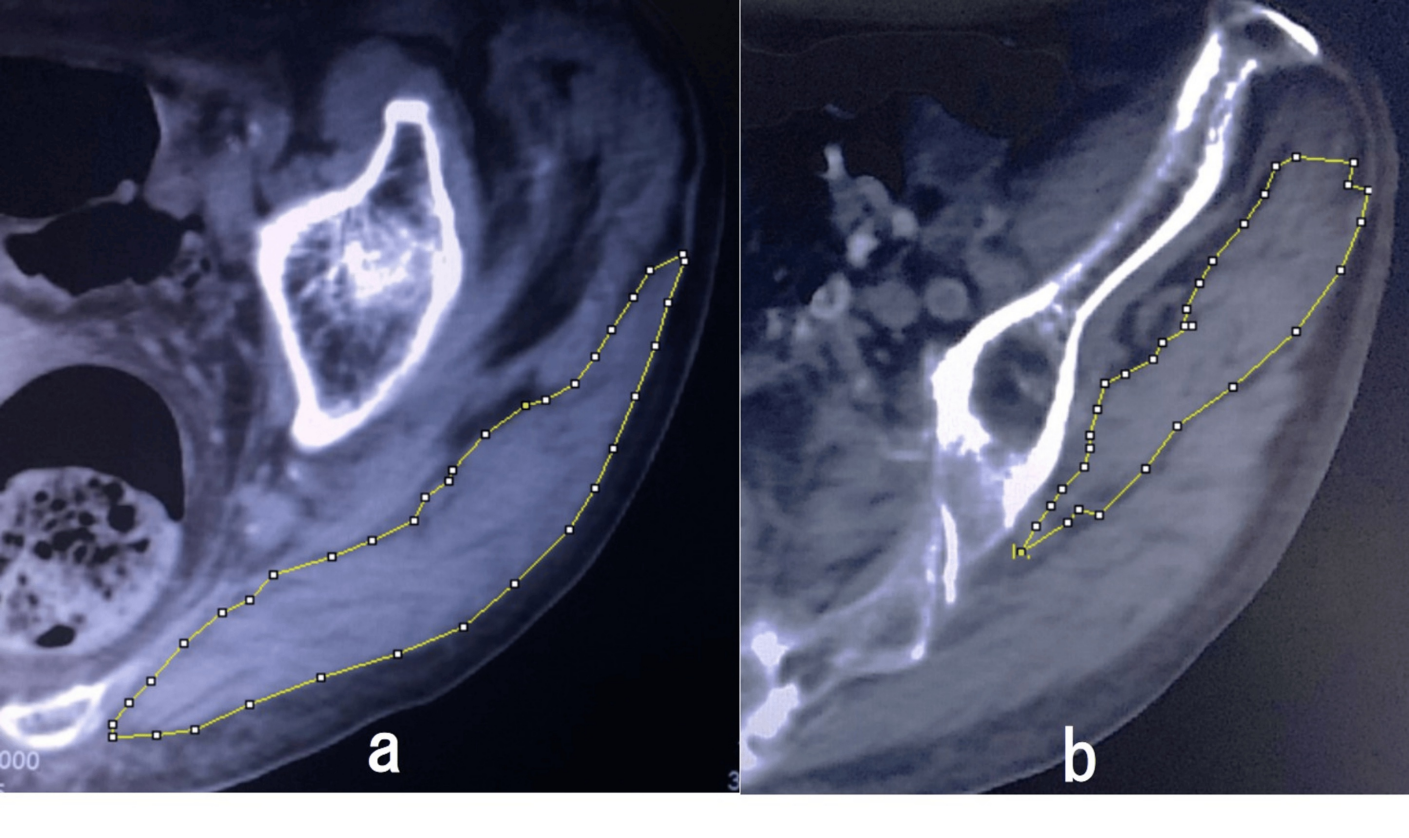
CT images of the gluteus maximus and medius muscles Panel (a) shows an axial image at the level of the greater trochanter's tip, highlighting the gluteus maximus. Panel (b) displays an axial image at the level of the third sacral vertebra, representing the gluteus medius.

**Figure 2:**
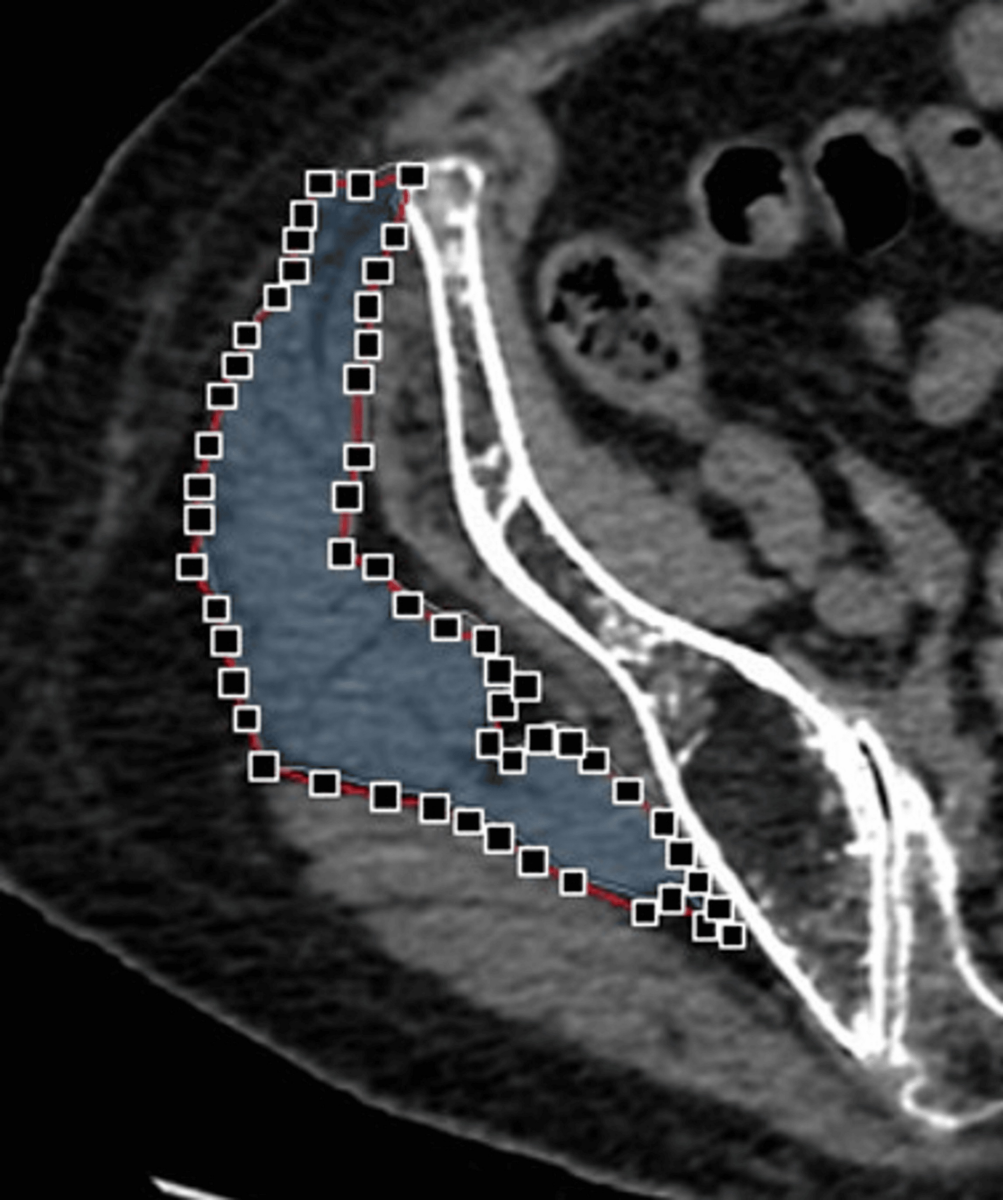
Cross-sectional area (CSA) of the gluteus medius muscle The boundaries of the muscle were manually traced by an observer, and the area was calculated based on the outlined contours.

Preoperative CT scans were performed from the iliac crest to below the lesser trochanter (120 kVp, Auto mA, Noise Index 9.5) with 5.0-mm thick slices at 55.0 mm intervals. The Optima CT 660 (GE Healthcare Japan, Tokyo, Japan) was used, and images were processed with AZE Virtual Place Fujin Raijin software (Version 3.0, AZE, Tokyo, Japan). Images were converted to digital imaging and communications in medicine (DICOM) format (512 × 512 pixels) for the quantification of CSA, bone, and fat to the nearest 0.01 cm². The non-injured side was used to obtain muscle data due to potential measurement interference from the fracture on the injured side. Axial images were selected for the gluteus maximus at the level of the greater trochanter tip and the gluteus medius at the level of the third sacral vertebra (Figure 1) [7,13]. The muscular border was manually traced and automatically calculated for the CSA and muscle density in HU (Figure 2).

The CSA and density in Hounsfield Units (HU) were evaluated on identical axial slices in images of both gluteus muscles. The density within the delineated muscular outline was calculated by segmenting into ranges for adipose tissue (-100 to -50 HU) and the higher value associated with muscle tissue. The total HU values for each tissue type are calculated after adjusting based on their proportional distribution within the region. Finally, a weighted HU density is computed to reflect the overall muscle composition, providing a measure of muscle quality and fat infiltration, as detailed in our preliminary study [14]. All measurements were conducted by a single examiner (M.N.).

Statistical analysis

Statistical analyses were performed using EZR software (Saitama Medical University Hospital, Saitama, Japan), a graphical user interface for R (The R Foundation for Statistical Computing, Vienna, Austria). Statistical significance was set at p < 0.05. Chi-square or Fisher’s exact tests were used to assess the significance of categorical variables, while Welch’s t-test and the Mann-Whitney U-test were used for numerical variables, depending on normality. Comparisons of CSA, muscle density in HU, and BMI-adjusted CSA between the two groups were performed.

## Results

Demographical details of each group

**Figure 3:**
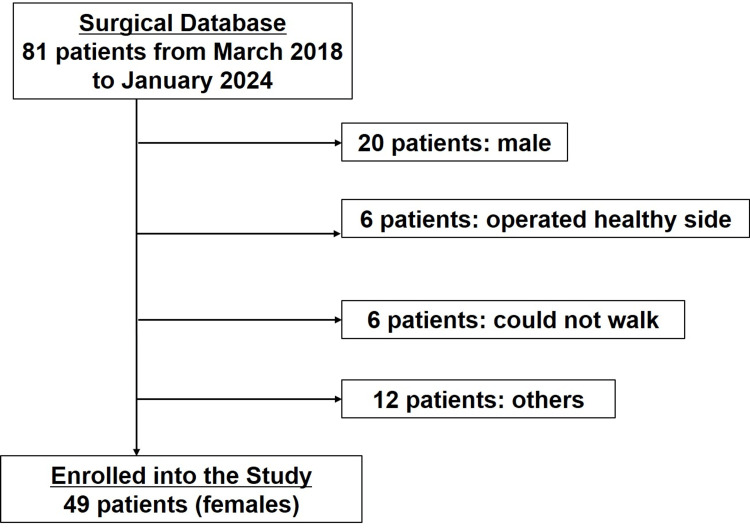
Flowchart of patient selection This flowchart illustrates the process of selecting patients for inclusion in the study, detailing the criteria applied and the number of patients at each stage.

Out of 81 patients, 49 patients aged 75 to 97 years (mean age: 87 years) met the inclusion criteria (Figure 3). The patients were divided into the ‘parallel bar group’ (n=19) and the ‘walker group’ (n=30) based on postoperative gait performance (Table 1). There were no significant differences between the groups in terms of age, body weight, height, or laterality of the affected side.

**Table 1:** Patient demographics and comparison of variables between the 'parallel bar' and 'walker' groups This table presents the demographic data of the patients, comparing age, body weight, height, and laterality of the affected side between the two groups. Statistical analysis was performed using Welch’s t-test for age, body weight, and height, and the chi-square test for laterality. Continuous variables are reported as means with standard deviations. Statistical significance between the two groups is denoted by an asterisk (*), indicating a significant difference with a p-value of less than 0.05 (p < 0.05). (^†^): Mean and standard deviation.

Variable	Parallel bar 19 patients	Walker 30 patients	p-value
Age group^†^ (years)	88.0±4.5	86.2±5.5	0.25
Body weight^†^ (kg)	42.0±8.8	44.1±7.7	0.41
Height^†^ (cm)	147.2±7.7	149.5±6.1	0.27
Laterality (number)	-	-	-
Right	7	16	0.175
Left	12	14	

CSA and density comparisons

The mean (and standard deviation (SD)) of CSA in the gluteus maximus was 2211.8 ± 469.8 mm² in the ‘parallel bar group’ and 2440.0 ± 586.2 mm² in the ‘walker group’ (p=0.15). The mean (and SD) CSA of the gluteus medius was 1751.7 ± 415.2 mm² in the ‘parallel bar group’ and 1869.1 ± 448.3 mm² in the ‘walker group’ (p=0.36) (Table 2).

**Table 2:** Comparison of CSA, muscle density, and BMI-adjusted CSA values between the 'parallel bar' and 'walker' groups The table provides the mean and standard deviation values for the gluteus maximus and medius in terms of CSA, muscle density (HU), and BMI-adjusted CSA. Statistical significance between the two groups is denoted by an asterisk (*), indicating a significant difference with a p-value of less than 0.05 (p < 0.05). (^†^): Mean and standard deviation. CSA: Cross-sectional area

Variable	Parallel bar 19 patients	Walker 30 patients	p-value
• CSA (mm^2^)	-	-	-
Glu. max^†^	2211.8±469.8	2440.0±586.2	0.15
Glu. med^†^	1751.7±415.2	1869.1±448.3	0.36
• Density	-	-	-
Glu. max^†^	13.6±12.7	20.6±13.0	0.07
Glu. med^†^	25.2±8.4	31.8±10.1	0.02*
• Adjusted.CSA	-	-	-
Glu. max^†^	116.4±26.8	124.3±29.2	0.35
Glu. med^†^	93.3±27.2	95.4±21.3	0.78

**Figure 4:**
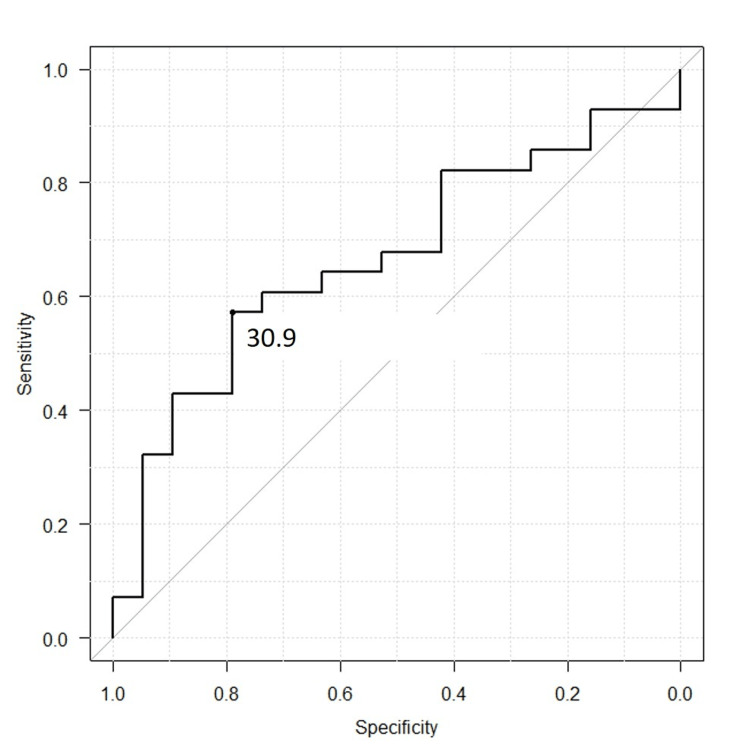
Receiver operating characteristic (ROC) curve illustrating the relationship between sensitivity and specificity The ROC curve demonstrates the balance between sensitivity and specificity for gluteus medius density in distinguishing between the 'parallel bar group' and the 'walker group.' The area under the curve (AUC: 0.719, 95% CI: 0.568-0.869) highlights the accuracy of gluteus medius density as a predictive factor. The optimal cut-off value, as determined by Youden's index, is 30.9 HU.

The mean (and SD) muscle density in HU for the gluteus maximus was 13.6 ± 12.7 HU in the ‘parallel bar group’ and 20.6 ± 13.0 HU in the ‘walker group’ (p=0.07). For the gluteus medius, the mean (and SD) density was 25.2 ± 8.4 HU in the ‘parallel bar group’ and 31.8 ± 10.1 HU in the ‘walker group’ (p=0.02). The receiver operating characteristic (ROC) curve identified a cut-off value of 30.9 HU for the gluteus medius, with sensitivity and specificity values of 60.7% and 78.9%, respectively (AUC: 0.719, 95% CI: 0.568-0.869) (Figure 4).

BMI-adjusted CSA comparison

The mean (and SD) BMI-adjusted CSA for the gluteus maximus was 116.4 ± 26.8 m²/10^6 ^kg in the ‘parallel bar group’ and 124.3 ± 29.2 m²/10^6^ kg in the ‘walker group’ (p=0.35). The mean BMI-adjusted CSA for the gluteus medius was 93.3 ± 27.2 m²/10^6^ kg in the ‘parallel bar group’ and 95.4 ± 21.3 m²/10^6^ kg in the ‘walker group’ (p=0.78).

Case presentation

**Figure 5:**
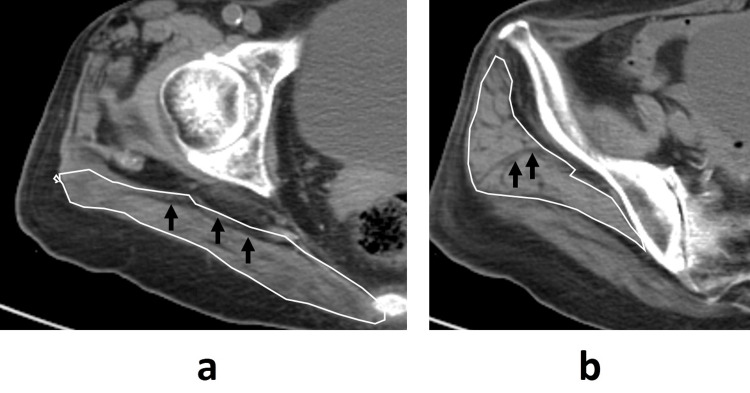
Case presentation of a 93-year-old female patient Panel (a) displays an axial image of the gluteus maximus, while Panel (b) shows the gluteus medius, with black arrows indicating intramuscular fat. This patient, who could walk with a cane before the injury, exhibited reduced muscle density of 9.8 HU in the gluteus maximus and 19.8 HU in the gluteus medius. Postoperatively, her walking ability was limited to the parallel bars, highlighting the significant impact of diminished muscle quality on gait performance.

A 93-year-old female patient sustained a left-sided pertrochanteric fracture due to a fall. Prior to the injury, she was able to walk with the aid of a cane. Axial CT images revealed that her gluteus maximus and medius had above-average CSAs of 2464.6 mm² and 2456.7 mm², respectively, with adjusted CSA values of 135.7 m²/10^6^ kg and 135.3 m²/10^6^ kg. However, her muscle density was notably low, with values of 9.8 HU in the gluteus maximus and 19.8 HU in the gluteus medius (Figure 5). Despite undergoing a consistent rehabilitation program, her postoperative walking ability remained limited to the use of a parallel bar.

## Discussion

The present study demonstrated that the mean and standard deviation of the gluteus medius muscle density in Hounsfield Units (HU) were significantly lower in the 'parallel bar group' (lower level of walking ability) at 25.2 ± 8.4 HU, compared to the 'walker group' (higher level) at 31.8 ± 10.1 HU (p=0.02). The difference in gluteus maximus density was only marginally insignificant (p=0.07). A critical cut-off value of 30.9 HU for the gluteus medius was identified as a predictor of the postoperative gait level. However, comparative analyses of the CSA and BMI-adjusted CSA revealed no statistically significant differences between the groups. These findings suggest that muscle density, particularly in the gluteus medius, may serve as a more reliable predictor of postoperative gait performance than CSA alone.

Muscular density as a decisive factor for walking

Muscle density emerged as a crucial indicator for predicting postoperative walking ability, whereas the CSA and adjusted CSA did not show significant predictive power. Previous research has indicated that muscle is a more sensitive and earlier marker of aging compared to bone, though the underlying mechanisms remain unclear [4,7,15]. Proximal femoral muscles, including the gluteus muscles, may be particularly vulnerable to myosteatosis, which can impair balance and mobility in older adults. Studies have emphasized the importance of muscle density, as it is associated with metabolic deficits and reduced physical activity in the elderly, worsening with age and inactivity [16]. Increased levels of myosteatosis have been observed in fall-prone individuals, particularly in the gluteus maximus and medius/minimus muscles [17].

A few studies have reported that the CSA adjusted for BMI is a significant factor influencing muscle strength, such as peak torque per body weight in the back flexors of female patients [17,18]. In this study, muscle strength was not targeted due to the challenges of accurately measuring it in the immediate postoperative period, where pain and weakness are prevalent. Instead, we propose a practical and reliable alternative: palpation of the gluteus muscles by experienced surgeons to assess volume and tension, providing an intuitive sense of muscle quality.

Modalities for quantifying muscle pathology

CT scans are among the most widely used imaging modalities for indirectly evaluating muscle pathology. While there is no universally accepted gold standard for quantifying myosteatosis or fat deposition within muscles, CT is effective for measuring both CSA and fatty infiltration, offering excellent reproducibility and reliability [17]. Thus, this method is suitable for both quantitative and qualitative muscle assessments [7]. Other imaging techniques, such as peripheral quantitative computed tomography, MRI, and quantitative ultrasound, also provide insights into muscle condition. Each modality has its advantages and disadvantages, including considerations of radiation exposure, technical complexity, resolution, cost, accessibility, and portability [5,17]. In this study, we utilized routine CT scans performed to assess fracture types, which allowed us to evaluate muscle condition without imposing additional burdens on patients.

Other factors related to gait ability

Several preoperative factors may influence postoperative walking ability, including age, dementia, pre-injury living situation, absence of a partner, anemia, electrolyte imbalances, lung function abnormalities, and chronic systemic diseases [2,19]. However, many of these factors are abstract or unreliable, often relying on patient or family recollections. Interestingly, the number of teeth has been linked to lower-extremity muscle strength-to-weight ratio two weeks postoperatively (p=0.04), suggesting that dental health might predict muscle strength and, consequently, physical function [4]. However, this factor is heavily influenced by dental hygiene practices, socioeconomic status, and educational background, introducing potential biases.

Strengths

This study has several strengths. First, all patients remained hospitalized in a single facility throughout the follow-up period, ensuring consistent rehabilitation under the direction of the attending physician familiar with each patient’s intraoperative findings and details. Second, the cohort was carefully selected with strict inclusion criteria regarding gender and age to minimize bias and ensure homogeneity, recognizing that demographic differences can significantly impact outcomes [7,20].

Limitations

This study also has limitations. First, the analysis was limited to univariate methods, rather than multivariate, which may reduce the overall significance of the findings. Second, although only one examiner measured the CSA and density, the simplicity of the measurement and the satisfactory inter-observer reliability in similar studies suggest that a single examiner would not substantially affect the results [14,21]. Third, the follow-up period was relatively short at only four weeks, though extending this period in elderly patients presents practical challenges. Fourth, the retrospective nature of the study meant that certain blood chemistry data, such as vitamin D levels, lipid profiles, and blood glucose, as well as lifestyle factors like smoking and alcohol consumption, which directly impact muscle mass and fat deposition, were not available [5,7,10]. Skeletal muscle is highly influenced by metabolic conditions, making these factors relevant [10].

Future directions

This study underscores the potential to enhance muscle quality assessment using CT images, building on prior research that has primarily focused on bone union or osseous structure in pertrochanteric fractures [22]. Future research should explore comprehensive rehabilitation programs tailored to patients with poor muscle quality. One approach is to continue strengthening exercises as before [16,21-23], while another approach may involve acknowledging the natural progression of muscle deterioration in the elderly, accepting that prolonged rehabilitation may be less effective. For instance, a 5.5-year follow-up study found that knee and hip strength in elderly women was associated with reduced mortality from all causes after adjusting for confounders [24]. These findings prompt a re-evaluation of treatment strategies. Additionally, further studies should aim to construct 3D muscle models, although many current studies, including ours, rely on single CT slices to represent muscle characteristics [25].

## Conclusions

This study examined preoperative CT images of women aged 75 years and older with pertrochanteric fractures, comparing the CSA and muscle density of the gluteal muscles between different levels of walking ability at four weeks postoperatively. The density of the gluteus medius in the superior gait group was significantly higher than in the inferior gait group, with a cut-off value of 30.9 HU. However, no statistically significant differences were found in the density of the gluteus maximus or CSA and BMI-adjusted CSA values for either muscle. These results partially support our hypothesis and highlight the importance of muscle evaluation in predicting gait performance.
